# Survey of ultrasound practice amongst podiatrists in the UK

**DOI:** 10.1186/s13047-018-0263-4

**Published:** 2018-05-24

**Authors:** Heidi J. Siddle, Aimie Patience, James Coughtrey, Jean Mooney, Martin Fox, Lindsey Cherry

**Affiliations:** 1 Leeds Institute of Rheumatic and Musculoskeletal Medicine, University of Leeds and Foot Health Department, Leeds Teaching Hospitals NHS Trust, 2nd Floor, Chapel Allerton Hospital, Chapeltown Road, Leeds, LS7 4SA UK; 2 Health and Life Science, Glasgow Caledonian University, Glasgow, UK; 3 Education Department, The College of Podiatry, London, UK; 4 Private Practitioner, Surrey, UK; 5 Manchester Leg Circulation Service, Pennine Acute Hospitals NHS Trust, Manchester, UK; 6 Health Sciences, University of Southampton, Southampton, UK

**Keywords:** Hand-held Doppler ultrasound, Mentorship, Podiatrists, Therapeutic ultrasound, Training, Ultrasound imaging

## Abstract

**Background:**

Ultrasound in podiatry practice encompasses musculoskeletal ultrasound imaging, vascular hand-held Doppler ultrasound and therapeutic ultrasound. Sonography practice is not regulated by the Health and Care Professions Council (HCPC), with no requirement to hold a formal qualification. The College of Podiatry does not currently define ultrasound training and competencies.

This study aimed to determine the current use of ultrasound, training received and mentorship received and/or provided by podiatrists using ultrasound.

**Methods:**

A quantitative study utilising a cross-sectional, on-line, single-event survey was undertaken within the UK.

**Results:**

Completed surveys were received from 284 podiatrists; 173 (70%) use ultrasound as part of their general practice, 139 (49%) for musculoskeletal problems, 131 (46%) for vascular assessment and 39 (14%) to support their surgical practice. Almost a quarter (*n* = 62) worked for more than one organisation; 202 (71%) were employed by the NHS and/or private sector (*n* = 118, 41%).

Nearly all (93%) respondents report using a hand-held vascular Doppler in their daily practice; 216 (82%) to support decisions regarding treatment options, 102 (39%) to provide diagnostic reports for other health professionals, and 34 (13%) to guide nerve blocks.

Ultrasound imaging was used by 104 (37%) respondents primarily to aid clinical decision making (*n* = 81) and guide interventions (steroid injections *n* = 67; nerve blocks *n* = 39). Ninety-three percent stated they use ultrasound imaging to treat their own patients, while others scan at the request of other podiatrists (*n* = 28) or health professionals (*n* = 18). Few use ultrasound imaging for research (*n* = 7) or education (*n* = 2).

Only 32 (11%) respondents (*n* = 20 private sector) use therapeutic ultrasound to treat patients presenting with musculoskeletal complaints, namely tendon pathologies.

Few respondents (18%) had completed formal post-graduate CASE (Consortium for the Accreditation of Sonographic Education) accredited ultrasound courses.

Forty (14%) respondents receive ultrasound mentorship; the majority from fellow podiatrists (*n* = 17) or medical colleagues (*n* = 15). Over half (*n* = 127) who do not have ultrasound mentorship indicated they would like a mentor predominantly for ultrasound imaging. Fifty-five (19%) report they currently provide ultrasound mentorship for others.

**Conclusions:**

Understanding the scope of ultrasound practice, the training undertaken and the requirements for mentorship will underpin the development of competencies and recommendations defined by the College of Podiatry to support professional development and ensure safe practice.

**Electronic supplementary material:**

The online version of this article (10.1186/s13047-018-0263-4) contains supplementary material, which is available to authorized users.

## Background

The use of ultrasound in podiatry practice encompasses ultrasound imaging (predominantly musculoskeletal), vascular hand-held Doppler ultrasound and therapeutic ultrasound. The College of Podiatry, Directorate of Podiatric Medicine has recognised the need to support members of the Society of Chiropodists and Podiatrists in developing and extending their scope of practice, with the appropriate competencies and recommendations in place. However, currently the scope of ultrasound practice amongst podiatrists in the UK, the training that they are undertaking and the requirements for mentorship to support professional development and safe practice are not fully understood.

Vascular assessment using hand held Doppler is used by the majority of podiatrists on a daily basis, as part of their routine patient assessment, particularly in the examination of patients with diabetes or those at risk of, or diagnosed as having peripheral arterial disease [1]. Diabetes UK and Foot in Diabetes UK (FDUK) in conjunction with the Society of Chiropodists and Podiatrists has published a competency framework which defines the levels of podiatric skills commensurate with the detection and diagnosis of the severity of peripheral vascular disease, by use of the hand-held Doppler and collaborative working practice [2]. This document differentiates between the skills and knowledge required at each level of competence ranging from the recent graduate to the consultant podiatrist.

Musculoskeletal ultrasound has evolved into an important method for identifying musculoskeletal abnormalities, confirming the diagnosis in patients with suspected inflammatory arthritis, monitoring therapeutic response, influencing clinical decision making and guiding interventions [3–6]. The role of the non-medical health professional has advanced with many health professionals, including podiatrists undertaking training and using ultrasound imaging to extend their scope of practice [7, 8] and as an outcome measure in research studies [9]. Podiatrists are performing ultrasound scans to support their clinical diagnosis, enhance treatment strategies and guide interventions for complex patients to improve the patient pathway [10]. In addition to undertaking training to develop their own skills, podiatrists with clinical expertise, as well as extensive knowledge of anatomy and experience, are also providing training for other health professionals and medical clinicians in ultrasound imaging [8].

Principles that underpin the safe use of therapeutic ultrasound [11] has formed part of the undergraduate podiatry curriculum in the UK for many years, and is used in podiatric musculoskeletal clinics for the treatment of a wide range of soft tissue injuries. The beneficial effects of ultrasound upon tissue may include increased blood flow, reduction in muscle spasm, increased extensibility of collagen fibres and a pro-inflammatory response [12]. In addition, pulsed ultrasound has been demonstrated to increase local blood flow through skin and soft tissues [13] thereby reducing pain and swelling, and promoting healing.

Unfortunately there is no legal requirement to hold a recognised ultrasound qualification in order to practice as a sonographer in the UK and sonography is not recognised as a profession by the Health and Care Professions Council (HCPC). Currently all formal training in the UK for ultrasound imaging is at a post graduate level (except for hand-held Doppler ultrasound) with most NHS Trusts recognising the need for an individual to undertake appropriate training, however this has not been defined and is not regulated. The UK College of Podiatry does not currently have guidance on ultrasound training for its members.

Undertaking this national survey provides an opportunity to identify current ultrasound practice and the training and mentorship undertaken by podiatrists in the UK. The survey will enhance the development of recommendations to support the education and training needs of podiatrists using ultrasound to ensure safe and appropriate practice for the benefit of patients. The survey will provide a national benchmark for the College of Podiatry to evaluate the effectiveness of those recommendations in supporting and developing ultrasound in podiatry practice in the future.

The primary aim of this study was, for the first time, to establish the current scope of ultrasound practice amongst podiatrists in the UK.

The specific objectives were to:

- Determine the current frequency of use of ultrasound imaging, vascular hand-held Doppler ultrasound and therapeutic ultrasound within podiatry practice.
- Determine the current training received by podiatrists to undertake the above investigations and/or interventions.
- Determine the current mentorship received and/or provided by podiatrists using ultrasound.

## Methods

Ethical approval was received from the University of Leeds, School of Medicine Research Ethics Committee (Ref No MREC15–107).

A quantitative study utilising a cross-sectional, on-line, single-event survey to elicit the scope of ultrasound practice and training undertaken amongst podiatrists was undertaken within the UK. The survey was developed utilising the research team’s experience in conducting research and survey design and in conjunction with the expertise of the College of Podiatry Ultrasound in Podiatry Specialist Advisory Group Committee. An iterative pilot phase was undertaken to test and refine the proposed questionnaire prior to the final version (Additional file 1). Members of the Ultrasound in Podiatry network were invited to complete the draft survey online to ensure face validity and usability.

An electronic survey technique was used (Bristol Online Surveys) to enable national completion. Podiatrists who were members of the Society of Chiropodists and Podiatrists (approximately 10,000) were invited to take part in the survey. The online survey was circulated to all members of the Society of Chiropodists and Podiatrists via the monthly professional e-newsletter, the Society professional publication (Podiatry Now), the College of Podiatry Specialist Advisory Groups (Ultrasound, MSK:UK, FDUK), the College of Podiatry Research Network, all the College of Podiatry Directorates and all Society associated social media sites (i.e. Twitter; Facebook). The survey was also promoted by the British Health Professionals in Rheumatology. The survey was open between 16th September 2016 and 30th November 2016, which enabled further promotion during the annual College of Podiatry conference.

A formal sample size calculation was not possible for this study. The distribution method was designed to maximise participation due to the currently unknown frequency of ultrasound use within this profession. The survey data was analysed using cross-tabulation and descriptive statistics.

## Results

Two hundred and eighty four people completed the online survey and all responses were complete and contained usable data. All respondents qualified in chiropody/podiatry between 1970 and 2016, with the majority qualifying between 1993 and 2003.

Almost a quarter of respondents (*n* = 62) work for more than one organisation; the majority (*n* = 202, 71%) are employed by the National Health Service (NHS) and/or private sector (*n* = 118, 41%). Others work in higher education institutions (HEI) (*n* = 18, 6%) or as Any Qualified Providers (AQPs) (*n* = 10, 3.5%). Respondents reported using various ultrasound modalities in their practice and for multiple reasons; 173 (70%) use ultrasound as part of their general practice, 139 (49%) for musculoskeletal problems, 131 (46%) for vascular/high risk assessment, 3 (1%) for podopaediatrics and 39 (14%) to support their surgical practice.

### Vascular hand held Doppler

Nearly all respondents use vascular hand-held Doppler in their clinical practice (93%) with over half using it daily (Table 1). It is used predominantly as a tool for assessing patients (*n* = 190), supporting decisions regarding treatment options (*n* = 216) and providing diagnostic reports for other health professionals (*n* = 101). Others reported using the hand-held Doppler to guide nerve blocks (*n* = 34) and corticosteroid injections (*n* = 5). Very few (*n* = 11) respondents use a vascular hand held Doppler for research purposes or as an educational tool (*n* = 1) (Table 2).

**Table 1:** Ultrasound practice by podiatrists in the UK

	Hand-held vascular Doppler (*n* = 262)	Ultrasound imaging (*n* = 104)	Therapeutic ultrasound (*n* = 32)
Daily	143	39	4
Weekly	72	45	16
Monthly	16	11	6
Less than Monthly	31	9	6

**Table 2:** Why podiatrists use ultrasound in their clinical practice

	Hand-held vascular Doppler	Ultrasound imaging	Therapeutic ultrasound
Treat own patients	252	97	32
Requested by other podiatrists	70	38	13
Requested by other health professionals	101	31	11
As a diagnostic service	41	12	2
Research	11	7	1
Education	1	2	1

### Ultrasound imaging

Ultrasound imaging is used by 37% of respondents (*n* = 104) in their clinical practice typically to guide steroid injections (*n* = 40) and nerve blocks (*n* = 24) in addition to aiding clinical decision making. Respondents typically use ultrasound imaging daily (*n* = 39) or weekly (*n* = 45) in their clinics. Most stated they use ultrasound imaging to treat their own patients (*n* = 97) while others scan at the request of other podiatrists (*n* = 28) or health professionals (*n* = 18) (Table 2). Again, comparatively few respondents use ultrasound imaging for research (*n* = 7) or educational purposes (*n* = 2).

### Therapeutic ultrasound

Only 32 respondents (11%) use therapeutic ultrasound in their weekly clinical practice; predominantly those working in the private sector (*n* = 20) (Tables 1 and 2). All stated they use therapeutic ultrasound to treat patients presenting with musculoskeletal complaints, namely tendon pathologies e.g. plantar fasciopathy or for the treatment of soft tissue masses e.g. Morton’s neuroma and bursitis. Twenty four respondents provide therapeutic ultrasound at the request of other podiatrists and health professionals (Table 2). Only one respondent reported using therapeutic ultrasound for conducting research and in higher education.

### Training

Fifty respondents (17%) had received no previous ultrasound training (Table 3). Almost 40% completed some form of ultrasound training as part of their undergraduate podiatry degree programme (*n* = 110) and a similar number had received in-house mentorship/supervision (*n* = 122). However comparatively few respondents had completed formal ultrasound courses. Unsurprisingly, on-line training had increased in popularity over the last 5 years (Table 4) and a similar trend could be found for the formal post-graduate CASE (Consortium for the Accreditation of Sonographic Education) accredited short/focus courses and CASE accredited full courses.

**Table 3:** Overview of the level of ultrasound training received by podiatrists

Level of ultrasound training undertaken by respondents	Number of respondents
None	50 (17.6%)
Pre-registration	110 (38.7%)
On line	13 (4.6%)
In house (mentorship)	112 (39.4%)
Ultrasound manufacturers’ course	44 (15.5%)
Organisation led	32 (11.3%)
CASE accredited short course	24 (8.4%)
CASE accredited full course	21 (7.4%)
Non-CASE accredited course	6 (2.1%)

**Table 4:** Number of years since podiatrists received training^a^

Level of ultrasound training	Years since completing ultrasound training					
	0–5	6–10	11–20	21–30	31–40	41–50
Pre-registration	29	14	33	17	9	1
On line	11	0	1	0	0	0
In house	71	24	12	4	0	0
Ultrasound manufacturers’ course	23	13	7	1	0	0
Organisation led	27	6	5	1	0	0
CASE accredited short course	23	1	0	0	0	0
CASE accredited full course	20	2	0	0	0	0
Non-CASE accredited course	5	0	1	0	0	0

^a^Not all respondents disclosed this information

### Mentorship

Around 15% of respondents currently receive ultrasound mentorship. Most mentors were podiatrists (*n* = 17) or medical practitioners (*n* = 11), however respondents also received mentorship from other allied health professionals and radiologists (Table 5). Of the 244 respondents who do not have ultrasound mentorship, over half indicated they would welcome the opportunity to have an ultrasound mentor (*n* = 127) predominantly for the use of ultrasound imaging. Almost 20% of respondents (*n* = 55) report they currently provide ultrasound mentorship for others; 34 for the use of hand-held vascular Doppler, 21 for ultrasound imaging and five provide mentorship for those using therapeutic ultrasound.

**Table 5:** Profession of respondents’ ultrasound mentor

Profession of current ultrasound mentor	Number of mentors
Medical	11
Podiatry	17
Other allied health professionals	7
Radiology	4
Ultrasound manufacturer	1

## Discussion

This on-line national survey has demonstrated that ultrasound practice amongst podiatrists in the UK includes the use of different modalities of ultrasound, in various clinical settings and for a range of purposes. Ultrasound is most commonly used as part of podiatrists’ general practice, in musculoskeletal services and for vascular assessment; it is most frequently used across NHS and private practice settings. Training continues to vary significantly across modalities with mentorship potentially being the most significant barrier to both undertaking training and maintaining competency.

Whilst the infrastructure of the Society of Chiropodists and Podiatrists provided an excellent means of contacting and recruiting podiatrists into this survey, information to confirm that participants were indeed members of the Society and/or practicing in the UK was inaccessible in the survey results. This should be considered a limitation of this study which could potentially impact on findings.

Despite the high level of usage of vascular hand-held Doppler in daily practice, more than half of the respondents reported that they either hadn’t undertaken any training or it was undertaken during their pre-registration degree (between 13 and 23 years ago). This raises concerns and further advocates the need for a competency framework to support on-going training in the use and subsequent interpretation of findings when using a hand-held Doppler as a tool for lower limb vascular assessment and risk status and/or prediction [2].

Where the use of vascular hand-held Doppler by podiatrists has been reviewed, it has been found in one study that ability to interpret different waveform signals was reasonable, although practitioners expressed concern over their own competence, citing a lack of specific knowledge, training or experience [14]. Another study has shown poor reliability around interpretation, when looking at both visual and audible waveforms; the need for clinicians to engage in regular and ongoing education was highlighted [15]. This issue is not specific to podiatrists, as shown by a study looking at variability amongst sonography professionals [16]. Considerable variation in waveform identification was found, from student to experienced physician to educator; 27% of triphasic, biphasic and monophasic waveforms were misidentified [16].

The importance of Doppler waveform interpretation is included in the minimum diagnostic assessment for peripheral arterial disease, along with history, symptom assessment and ankle brachial pressure index testing. With over 20% of the population aged 60+ having peripheral arterial disease, the need to diagnose it accurately and early has been highlighted by a National Institute for Health and Clinical Excellence (NICE) Guideline (CG 147) [17] and subsequent NICE Quality Standard (QS52) [18]. By palpating and interpreting foot pulses and arterial flow at assessment and reviews, all podiatrists play a vital role in the early diagnosis of this serious disease, with the opportunity to ensure best treatment is commenced. There is an identified need to optimise Doppler interpretation knowledge and skills via competency-based training and from the existing studies published on reliability and interpretation with Doppler waveforms. This appears to be needed for all podiatrists working in general practice and across specialties.

Just over a third of respondents use musculoskeletal ultrasound imaging in their daily or weekly practice to inform treatment through aiding clinical decision making and guiding interventions. The absence of a legal requirement to hold a recognised ultrasound qualification in order to practice as a sonographer in the UK might be the reason that only 18% of those using ultrasound imaging have completed a formal CASE accredited post-graduate course, which includes competency assessment. However, these courses require the podiatrist to have a mentor and this survey has identified that over half of respondents would like a mentor for ultrasound imaging, which could potentially be a barrier to undertaking CASE accredited courses.

The College of Podiatry has recently become a member organisation of CASE who undertake the accreditation of high quality training programmes and focused courses. This includes the accreditation of foot and ankle specific courses that promote best ultrasound practice and ensure that ultrasound practitioners, including podiatrists are safe and competent to practise in order to maximise the benefits to patients. However, the findings from this survey highlight the lack of mentorship available, a persistent difficulty acknowledged by those wishing to undertake formal training. Whilst needing to address the guidance on ultrasound training for podiatrists, it will be equally important to develop a sustainable mentorship programme to ensure those who want to embark on formal training courses are able to meet the entry requirements of having a mentor.

Although therapeutic ultrasound has traditionally been taught at undergraduate level, the role of therapeutic ultrasound appears to be diminishing in podiatry practice. Further understanding of the training requirements and process for competency assessment is required to determine the future role it has to play in podiatry practice.

The findings from this survey indicate that all modalities of ultrasound are rarely used by podiatrists in research and education. However, recent European recommendations for non-medical health professionals, including podiatrists using musculoskeletal ultrasound imaging, advocate that health professionals may use musculoskeletal ultrasound as a tool for research including health professional-led studies [8]. Ultrasound is being introduced into medical and health professional education programmes to increase the understanding of anatomy and guide interventions such as obtaining vascular access and giving local anaesthesia and steroid injections. The College of Podiatry Core Curriculum for Podiatric Medicine states that competence should be reached in using a hand-held Doppler for undertaking evidence based patient assessments and that diagnostic ultrasound is an area of advanced practice which may be deemed ‘core’ in the future [19]. Hence, further understanding of when and how education providers are currently using or teaching ultrasound will increase our understanding of how ultrasound is likely to be used by podiatrists in the future. It may also offer the opportunity to guide the development of undergraduate and post-graduate education programmes.

## Conclusions

This nationwide survey has, for the first time, provided an understanding of the scope of ultrasound practice amongst podiatrists in the UK. The survey results demonstrated the limited training that underpins current practice and highlighted the requirements for mentorship to support professional progression. The skills of those podiatrists who are using ultrasound are recognised by their peers and other professionals alike, and act as sources for referral and education. These findings will underpin the development of competencies and recommendations defined by the College of Podiatry, to support professional development and ensure safe practice of ultrasound in the UK.

## Additional file

Additional file 1: Ultrasound in podiatry online survey questions. (PDF 384 kb)

## Abbreviations

AQP: Any Qualified Provider
CASE: Consortium for the Accreditation of Sonographic Education
FDUK: Foot in Diabetes UK
HCPC: Health and Care Professions Council
HEI: Higher education institutions
NHS: National Health Service
NICE: National Institute for Health and Clinical Excellence
