# Supplementary material for: Survey of ultrasound practice amongst podiatrists in the UK
Source: J Foot Ankle Res. 2018 May 24;11:18. doi: 10.1186/s13047-018-0263-4 (PMC5968714; doi:10.1186/s13047-018-0263-4)
Supplement: Supplementary file 1 — Ultrasound in podiatry online survey questions. (PDF 384 kb) [file 13047_2018_263_MOESM1_ESM.pdf]

Ultrasound in Podiatry

Page 1: Survey of ultrasound practice amongst podiatrists in the UK

The use of ultrasound in podiatry practice encompasses ultrasound imaging (predominantly musculoskeletal), vascular hand-held Doppler ultrasound and therapeutic ultrasound. The College of Podiatry, Directorate of Podiatric Medicine has recognised the need to support to members of the Society of Chiropodists and Podiatrists in developing and extending their scope of practice, with the appropriate competencies and recommendations in place. However, currently we do not fully understand the scope of ultrasound practice amongst podiatrists in the UK, the training that they are undertaking and the requirements for mentorship to support professional development and safe practice.

Undertaking this national survey provides an opportunity to identify current ultrasound practice and the training and mentorship undertaken by podiatrists. The survey will enhance the development of recommendations to support the education and training needs of podiatrists using ultrasound to ensure safe and appropriate practice for the benefit of patients. The survey will provide a benchmark for the College of Podiatry to evaluate the effectiveness of recommendations for the use ultrasound in podiatry in the future.

Page 2: Ultrasound in Podiatry Survey: Participant Information Sheet

What is the purpose of the study?

The aim of this study is to understand the current frequency of use of ultrasound imaging, vascular hand-held Doppler ultrasound and therapeutic ultrasound within podiatry practice. The study also aims to determine the training received by podiatrists to undertake the above investigations and/or interventions and the mentorship received and/or provided by podiatrists using ultrasound.

Why have I been chosen?

You have been chosen because you are a podiatrist and a member of the Society of Chiropodists and Podiatrists.

Do you have to take part?

Taking part in this study is entirely voluntary, it is up to you to decide whether or not to take part and if you do not wish to do so, you do not have to give a reason. If you begin the survey and decide to stop, you are free to do so at any point. Partially completed questionnaires will not be submitted. Completed surveys cannot be withdrawn once they are submitted.

What will happen to me if I take part?

If you decide to take part, you only need to complete this online survey. You will not be contacted again.

Will my taking part in this study be kept confidential?

Your participation in this survey will be completely anonymous.

What will happen to the results of the study?

The results of this survey will enable us to establish the current scope of ultrasound practice at this point in time within the UK. This information will be used to ensure that the College of Podiatry Ultrasound Advisory Group support members in developing their practice. The

survey will also provide a benchmark of where the profession is currently and allow us to measure the impact of the College of Podiatry Ultrasound in Podiatry Specialist Advisory Group in the future. The results are likely to be published in a medical journal and presented at conferences but you will not be identified in any publication or presentation.

Who is organising and funding the study?

This study has been instigated by Dr Heidi Siddle, University of Leeds and Dr Lindsey Cherry, University of Southampton on behalf of the College of Podiatry Ultrasound in Podiatry Specialist Advisory Group Committee. The study has not received any direct funding but the study is being supported by the College of Podiatry.

Who has reviewed this study?

This study has been independently reviewed and ethical approval sought from the University of Leeds, School of Medicine Research Ethics Committee (SoMREC project number MREC 15-107).

Further information and contact details:

For further information about this study please contact Dr Heidi Siddle, h.siddle@leeds.ac.uk , Dr Lindsey Cherry, l.cherry@soton.ac.uk or Mr James Coughtrey, JC@scpod.org

1. I confirm that I have read and understand the participant information sheet. I understand that my participation is voluntary and that I am free to withdraw at any time without submitting the online survey. I understand that my responses cannot be withdrawn once they are submitted. ✱ *Required*

- ○ Yes

Page 3: Clinical Role

2. When did you qualify as a Chiropodist or Podiatrist? Please state which year. ✱ *Required*

3. In which clinical area do you work? (please select all that apply) ✱ *Required*

- ☐ Diabetes
- ☐ Musculoskeletal care
- ☐ Wound care
- ☐ Orthopaedics
- ☐ Podiatric surgery
- ☐ Rheumatology
- ☐ General practice
- ☐ Other

3.a. If you selected Other, please specify:

Page 4: Ultrasound Practice

4. In which organisational setting do you work? (please select all that apply) ✱ *Required*

- [ ] The National Health Service (NHS)
- [ ] An independent ‘any qualified provider’ (AQP) practice
- [ ] Private practice
- [ ] A university or other higher education institution (HEI)
- [ ] Other

4.a. If you selected Other, please specify:

Please note that you will be asked about your ultrasound practice in each organisational setting that you work in.

4.b. What type of ultrasound do you use in your NHS practice? (please select all that apply)

- [ ] Ultrasound Imaging
- [ ] Vascular hand-held Doppler ultrasound
- [ ] Therapeutic ultrasound
- [ ] Other
- [ ] Not applicable

4.b.i. If you selected Other, please specify:

4.b.ii. Please estimate how often you use ultrasound imaging in your NHS practice.

- ☐ Daily
- ☐ Weekly
- ☐ Monthly
- ☐ Less frequently than once a month
- ☐ Other

4.b.ii.a. If you selected Other, please specify:

4.b.iii. For what do you use ultrasound imaging in your NHS practice?

- ☐ As part of my routine initial patient assessment
- ☐ To aid clinical decision making
- ☐ To guide steroid injections
- ☐ To guide nerve block injections
- ☐ To provide diagnostic reports to other health professionals
- ☐ Other

4.b.iii.a. If you selected Other, please specify:

4.b.iv. In your NHS practice do you undertake ultrasound imaging . . . (please select all that apply)

- ☐ to treat your own patients?
- ☐ at the request of other podiatrists?
- ☐ at the request of other health professionals?
- ☐ as an independent diagnostic service?
- ☐ for clinical research?
- ☐ Other

4.b.iv.a. If you selected Other, please specify:

4.b.v. Please estimate how often you use vascular hand-held Doppler ultrasound in your NHS practice

- ☐ Daily
- ☐ Weekly
- ☐ Monthly
- ☐ Less frequently than once a month
- ☐ Other

4.b.v.a. If you selected Other, please specify:

4.b.vi. For what do you use vascular hand-held Doppler ultrasound in your NHS practice?

- ☐ As part of my routine initial patient assessment
- ☐ To aid clinical decision making

- [ ] To guide steroid injections
- [ ] To guide nerve block injections
- [ ] To provide diagnostic reports to other health professionals
- [ ] Other

4.b.vi.a. If you selected Other, please specify:

[ ]

4.b.vii. In your NHS practice do you undertake vascular hand-held Doppler ultrasound . . . (please select all that apply)

- [ ] to treat your own patients?
- [ ] at the request of other podiatrists?
- [ ] at the request of other health professionals?
- [ ] as an independent diagnostic service?
- [ ] for clinical research?
- [ ] Other

4.b.vii.a. If you selected Other, please specify:

[ ]

4.b.viii. Please estimate how often you use therapeutic ultrasound in your NHS practice

- [ ] Daily
- [ ] Weekly
- [ ] Monthly

- [ ] Less frequently than once a month
- [ ] Other

4.b.viii.a. If you selected Other, please specify:

4.b.ix. For what do you use therapeutic ultrasound in your NHS practice?

4.b.x. In your NHS practice do you undertake therapeutic ultrasound . . . (please select all that apply)

- [ ] to treat your own patients?
- [ ] at the request of other podiatrists?
- [ ] at the request of other health professionals?
- [ ] as an independent diagnostic service?
- [ ] for clinical research?
- [ ] Other

4.b.x.a. If you selected Other, please specify:

4.b.xi. Please estimate how often you use other ultrasound in your NHS practice

- [ ] Daily
- [ ] Weekly
- [ ] Monthly
- [ ] Less frequently than once a month
- [ ] Other

4.b.xi.a. If you selected Other, please specify:

4.b.xii. For what do you use other ultrasound in your NHS practice?

4.b.xiii. In your NHS practice do you undertake other ultrasound . . . (please select all that apply)

- [ ] to treat your own patients?
- [ ] at the request of other podiatrists?
- [ ] at the request of other health professionals?
- [ ] as an independent diagnostic service?
- [ ] for clinical research?
- [ ] Other

4.b.xiii.a. If you selected Other, please specify:

4.c. What type of ultrasound do you use in your AQP practice? (please select all that apply)

- ☐ Ultrasound Imaging
- ☐ Vascular hand-held Doppler ultrasound
- ☐ Therapeutic ultrasound
- ☐ Other
- ☐ Not applicable

4.c.i. If you selected Other, please specify:

4.c.ii. Please estimate how often you use ultrasound imaging in your AQP practice.

- ☐ Daily
- ☐ Weekly
- ☐ Monthly
- ☐ Less frequently than once a month
- ☐ Other

4.c.ii.a. If you selected Other, please specify:

4.c.iii. For what do you use ultrasound imaging in your AQP practice?

- [ ] As part of my routine initial patient assessment
- [ ] To aid clinical decision making
- [ ] To guide steroid injections
- [ ] To guide nerve block injections
- [ ] To provide diagnostic reports to other health professionals
- [ ] Other

4.c.iii.a. If you selected Other, please specify:

4.c.iv. In your AQP practice do you undertake ultrasound imaging . . . (please select all that apply)

- [ ] to treat your own patients?
- [ ] at the request of other podiatrists?
- [ ] at the request of other health professionals?
- [ ] as an independent diagnostic service?
- [ ] for clinical research?
- [ ] Other

4.c.iv.a. If you selected Other, please specify:

4.c.v. Please estimate how often you use vascular hand-held Doppler ultrasound in your

AQP practice

- ☐ Daily
- ☐ Weekly
- ☐ Monthly
- ☐ Less frequently than once a month
- ☐ Other

4.c.v.a. If you selected Other, please specify:

4.c.vi. For what do you use vascular hand-held Doppler ultrasound in your AQP practice?

- ☐ As part of my routine initial patient assessment
- ☐ To aid clinical decision making
- ☐ To guide steroid injections
- ☐ To guide nerve block injections
- ☐ To provide diagnostic reports to other health professionals
- ☐ Other

4.c.vi.a. If you selected Other, please specify:

4.c.vii. In your AQP practice do you undertake vascular hand-held Doppler ultrasound . . . (please select all that apply)

- [ ] to treat your own patients?
- [ ] at the request of other podiatrists?
- [ ] at the request of other health professionals?
- [ ] as an independent diagnostic service?
- [ ] for clinical research?
- [ ] Other

4.c.vii.a. If you selected Other, please specify:

4.c.viii. Please estimate how often you use therapeutic ultrasound in your AQP practice

- [ ] Daily
- [ ] Weekly
- [ ] Monthly
- [ ] Less frequently than once a month
- [ ] Other

4.c.viii.a. If you selected Other, please specify:

4.c.ix. For what do you use therapeutic ultrasound in your AQP practice?

4.c.x. In your AQP practice do you undertake therapeutic ultrasound . . . (please select all that apply)

- ☐ to treat your own patients?
- ☐ at the request of other podiatrists?
- ☐ at the request of other health professionals?
- ☐ as an independent diagnostic service?
- ☐ for clinical research?
- ☐ Other

4.c.x.a. If you selected Other, please specify:

4.c.xi. Please estimate how often you use other ultrasound in your AQP practice

- ☐ Daily
- ☐ Weekly
- ☐ Monthly
- ☐ Less frequently than once a month
- ☐ Other

4.c.xi.a. If you selected Other, please specify:

4.c.xii. For what do you use other ultrasound in your AQP practice?

4.c.xiii. In your AQP practice do you undertake other ultrasound . . . (please select all that apply)

- ☐ to treat your own patients?
- ☐ at the request of other podiatrists?
- ☐ at the request of other health professionals?
- ☐ as an independent diagnostic service?
- ☐ for clinical research?
- ☐ Other

4.c.xiii.a. If you selected Other, please specify:

4.d. What type of ultrasound do you use in your private practice? (please select all that apply)

- ☐ Ultrasound Imaging
- ☐ Vascular hand-held Doppler ultrasound
- ☐ Therapeutic ultrasound
- ☐ Other
- ☐ Not applicable

4.d.i. If you selected Other, please specify:

4.d.ii. Please estimate how often you use ultrasound imaging in your private practice.

- ☐ Daily
- ☐ Weekly
- ☐ Monthly
- ☐ Less frequently than once a month
- ☐ Other

4.d.ii.a. If you selected Other, please specify:

4.d.iii. For what do you use ultrasound imaging in your private practice?

- ☐ As part of my routine initial patient assessment
- ☐ To aid clinical decision making
- ☐ To guide steroid injections
- ☐ To guide nerve block injections
- ☐ To provide diagnostic reports to other health professionals
- ☐ Other

4.d.iii.a. If you selected Other, please specify:

4.d.iv. In your private practice do you undertake ultrasound imaging . . . (please select all that apply)

- ☐ to treat your own patients?
- ☐ at the request of other podiatrists?
- ☐ at the request of other health professionals?
- ☐ as an independent diagnostic service?
- ☐ for clinical research?
- ☐ Other

4.d.iv.a. If you selected Other, please specify:

4.d.v. Please estimate how often you use vascular hand-held Doppler ultrasound in your private practice

- ☐ Daily
- ☐ Weekly
- ☐ Monthly
- ☐ Less frequently than once a month
- ☐ Other

4.d.v.a. If you selected Other, please specify:

4.d.vi. For what do you use vascular hand-held Doppler ultrasound in your private practice?

- ☐ As part of my routine initial patient assessment
- ☐ To aid clinical decision making
- ☐ To guide steroid injections
- ☐ To guide nerve block injections
- ☐ To provide diagnostic reports to other health professionals
- ☐ Other

4.d.vi.a. If you selected Other, please specify:

4.d.vii. In your private practice do you undertake vascular hand-held Doppler ultrasound . . . (please select all that apply)

- ☐ to treat your own patients?
- ☐ at the request of other podiatrists?
- ☐ at the request of other health professionals?
- ☐ as an independent diagnostic service?
- ☐ for clinical research?
- ☐ Other

4.d.vii.a. If you selected Other, please specify:

4.d.viii. Please estimate how often you use therapeutic ultrasound in your private practice

- [ ] Daily
- [ ] Weekly
- [ ] Monthly
- [ ] Less frequently than once a month
- [ ] Other

4.d.viii.a. If you selected Other, please specify:

4.d.ix. For what do you use therapeutic ultrasound in your private practice?

4.d.x. In your private practice do you undertake therapeutic ultrasound . . . (please select all that apply)

- [ ] to treat your own patients?
- [ ] at the request of other podiatrists?
- [ ] at the request of other health professionals?
- [ ] as an independent diagnostic service?
- [ ] for clinical research?
- [ ] Other

4.d.x.a. If you selected Other, please specify:

4.d.xi. Please estimate how often you use other ultrasound in your private practice

- ☐ Daily
- ☐ Weekly
- ☐ Monthly
- ☐ Less frequently than once a month
- ☐ Other

4.d.xi.a. If you selected Other, please specify:

4.d.xii. For what do you use other ultrasound in your private practice?

4.d.xiii. In your private practice do you undertake other ultrasound . . . (please select all that apply)

- ☐ to treat your own patients?
- ☐ at the request of other podiatrists?
- ☐ at the request of other health professionals?
- ☐ as an independent diagnostic service?
- ☐ for clinical research?

- [ ] Other

4.d.xiii.a. If you selected Other, please specify:

4.e. What type of ultrasound do you use in your university or other HEI role? (please select all that apply)

- [ ] Ultrasound Imaging
- [ ] Vascular hand-held Doppler ultrasound
- [ ] Therapeutic ultrasound
- [ ] Other
- [ ] Not applicable

4.e.i. If you selected Other, please specify:

4.e.ii. Please estimate how often you use ultrasound imaging in your university or other HEI role.

- [ ] Daily
- [ ] Weekly
- [ ] Monthly
- [ ] Less frequently than once a month
- [ ] Other

4.e.ii.a. If you selected Other, please specify:

4.e.iii. For what do you use ultrasound imaging in your university or HEI role?

- [ ] As part of my routine initial patient assessment
- [ ] To aid clinical decision making
- [ ] To guide steroid injections
- [ ] To guide nerve block injections
- [ ] To provide diagnostic reports to other health professionals
- [ ] Other

4.e.iii.a. If you selected Other, please specify:

4.e.iv. In your university or other HEI role do you undertake ultrasound imaging . . . (please select all that apply)

- [ ] to treat your own patients?
- [ ] at the request of other podiatrists?
- [ ] at the request of other health professionals?
- [ ] as an independent diagnostic service?
- [ ] for clinical research?
- [ ] Other

4.e.iv.a. If you selected Other, please specify:

4.e.v. Please estimate how often you use vascular hand-held Doppler ultrasound in your university or other HEI role

- ☐ Daily
- ☐ Weekly
- ☐ Monthly
- ☐ Less frequently than once a month
- ☐ Other

4.e.v.a. If you selected Other, please specify:

4.e.vi. For what do you use vascular hand-held Doppler ultrasound in your university or other HEI role?

- ☐ As part of my routine initial patient assessment
- ☐ To aid clinical decision making
- ☐ To guide steroid injections
- ☐ To guide nerve block injections
- ☐ To provide diagnostic reports to other health professionals
- ☐ Other

4.e.vi.a. If you selected Other, please specify:

4.e.vii. In your university or other HEI role do you undertake vascular hand-held Doppler ultrasound . . . (please select all that apply)

- ☐ to treat your own patients?
- ☐ at the request of other podiatrists?
- ☐ at the request of other health professionals?
- ☐ as an independent diagnostic service?
- ☐ for clinical research?
- ☐ Other

4.e.vii.a. If you selected Other, please specify:

4.e.viii. Please estimate how often you use therapeutic ultrasound in your university or other HEI role

- ☐ Daily
- ☐ Weekly
- ☐ Monthly
- ☐ Less frequently than once a month
- ☐ Other

4.e.viii.a. If you selected Other, please specify:

4.e.ix. For what do you use therapeutic ultrasound in your university or other HEI role?

4.e.x. In your university or other HEI role do you undertake therapeutic ultrasound . . . (please select all that apply)

- ☐ to treat your own patients?
- ☐ at the request of other podiatrists?
- ☐ at the request of other health professionals?
- ☐ as an independent diagnostic service?
- ☐ for clinical research?
- ☐ Other

4.e.x.a. If you selected Other, please specify:

4.e.xi. Please estimate how often you use other ultrasound in your university or other HEI role

- ☐ Daily
- ☐ Weekly
- ☐ Monthly

- ☐ Less frequently than once a month
- ☐ Other

4.e.xi.a. If you selected Other, please specify:

4.e.xii. For what do you use other ultrasound in your university or other HEI role?

4.e.xiii. In your university or other HEI role do you undertake other ultrasound . . . (please select all that apply)

- ☐ to treat your own patients?
- ☐ at the request of other podiatrists?
- ☐ at the request of other health professionals?
- ☐ as an independent diagnostic service?
- ☐ for clinical research?
- ☐ Other

4.e.xiii.a. If you selected Other, please specify:

4.f. What type of ultrasound do you use in other organisational settings? (please select all that

apply)

- ☐ Ultrasound Imaging
- ☐ Vascular hand-held Doppler ultrasound
- ☐ Therapeutic ultrasound
- ☐ Other
- ☐ Not applicable

4.f.i. If you selected Other, please specify:

4.f.ii. Please estimate how often you use ultrasound imaging in your practice in other organisational settings.

- ☐ Daily
- ☐ Weekly
- ☐ Monthly
- ☐ Less frequently than once a month
- ☐ Other

4.f.ii.a. If you selected Other, please specify:

4.f.iii. For what do you use ultrasound imaging in your practice in other organisational settings?

- ☐ As part of my routine initial patient assessment

- [ ] To aid clinical decision making
- [ ] To guide steroid injections
- [ ] To guide nerve block injections
- [ ] To provide diagnostic reports to other health professionals
- [ ] Other

4.f.iii.a. If you selected Other, please specify:

4.f.iv. In your practice in other organisational settings do you undertake ultrasound imaging . . . (please select all that apply)

- [ ] to treat your own patients?
- [ ] at the request of other podiatrists?
- [ ] at the request of other health professionals?
- [ ] as an independent diagnostic service?
- [ ] for clinical research?
- [ ] Other

4.f.iv.a. If you selected Other, please specify:

4.f.v. Please estimate how often you use vascular hand-held Doppler ultrasound in your practice in other organisational settings

- [ ] Daily

- [ ] Weekly
- [ ] Monthly
- [ ] Less frequently than once a month
- [ ] Other

4.f.v.a. If you selected Other, please specify:

4.f.vi. For what do you use vascular hand-held Doppler ultrasound in your practice in other organisational settings?

- [ ] As part of my routine initial patient assessment
- [ ] To aid clinical decision making
- [ ] To guide steroid injections
- [ ] To guide nerve block injections
- [ ] To provide diagnostic reports to other health professionals
- [ ] Other

4.f.vi.a. If you selected Other, please specify:

4.f.vii. In your practice in other organisational settings do you undertake vascular hand-held Doppler ultrasound . . . (please select all that apply)

- [ ] to treat your own patients?
- [ ] at the request of other podiatrists?

- [ ] at the request of other health professionals?
- [ ] as an independent diagnostic service?
- [ ] for clinical research?
- [ ] Other

4.f.vii.a. If you selected Other, please specify:

4.f.viii. Please estimate how often you use therapeutic ultrasound in your practice in other organisational settings

- [ ] Daily
- [ ] Weekly
- [ ] Monthly
- [ ] Less frequently than once a month
- [ ] Other

4.f.viii.a. If you selected Other, please specify:

4.f.ix. For what do you use therapeutic ultrasound in your practice in other organisational settings?

4.f.x. In your practice in other organisational settings do you undertake therapeutic ultrasound . . . (please select all that apply)

- ☐ to treat your own patients?
- ☐ at the request of other podiatrists?
- ☐ at the request of other health professionals?
- ☐ as an independent diagnostic service?
- ☐ for clinical research?
- ☐ Other

4.f.x.a. If you selected Other, please specify:

4.f.xi. Please estimate how often you use other ultrasound in your practice in other organisational settings

- ☐ Daily
- ☐ Weekly
- ☐ Monthly
- ☐ Less frequently than once a month
- ☐ Other

4.f.xi.a. If you selected Other, please specify:

4.f.xii. For what do you use other ultrasound in your practice in other organisational settings?

4.f.xiii. In your practice in other organisational settings do you undertake other ultrasound . . . (please select all that apply)

- ☐ to treat your own patients?
- ☐ at the request of other podiatrists?
- ☐ at the request of other health professionals?
- ☐ as an independent diagnostic service?
- ☐ for clinical research?
- ☐ Other

4.f.xiii.a. If you selected Other, please specify:

4.g. Please indicate the whole time equivalent (WTE) of a week that you spend working in an 'other' organisation

- ☐ 0.1 WTE
- ☐ 0.2 WTE
- ☐ 0.3 WTE
- ☐ 0.4 WTE
- ☐ 0.5 WTE
- ☐ 0.6 WTE
- ☐ 0.7 WTE
- ☐ 0.8 WTE
- ☐ 0.9 WTE

- [ ] 1.0 WTE

4.h. Please indicate the whole time equivalent (WTE) of a week that you spend working in a University or other Higher Education Institution

- [ ] 0.1 WTE
- [ ] 0.2 WTE
- [ ] 0.3 WTE
- [ ] 0.4 WTE
- [ ] 0.5 WTE
- [ ] 0.6 WTE
- [ ] 0.7 WTE
- [ ] 0.8 WTE
- [ ] 0.9 WTE
- [ ] 1.0 WTE

4.i. Please indicate the whole time equivalent (WTE) of a week that you spend working in a private practice

- [ ] 0.1 WTE
- [ ] 0.2 WTE
- [ ] 0.3 WTE
- [ ] 0.4 WTE
- [ ] 0.5 WTE
- [ ] 0.6 WTE
- [ ] 0.7 WTE
- [ ] 0.8 WTE
- [ ] 0.9 WTE
- [ ] 1.0 WTE

4.j. Please indicate the whole time equivalent (WTE) of a week that you spend working in an independent ‘any qualified provider’ practice

- [ ] 0.1 WTE
- [ ] 0.2 WTE
- [ ] 0.3 WTE
- [ ] 0.4 WTE
- [ ] 0.5WTE
- [ ] 0.6 WTE
- [ ] 0.7 WTE
- [ ] 0.8 WTE
- [ ] 0.9 WTE
- [ ] 1.0 WTE

4.k. Please indicate the whole time equivalent (WTE) of a week that you spend working in the National Health Service

- [ ] 0.1 WTE
- [ ] 0.2 WTE
- [ ] 0.3 WTE
- [ ] 0.4 WTE
- [ ] 0.5 WTE
- [ ] 0.6 WTE
- [ ] 0.7 WTE
- [ ] 0.8 WTE
- [ ] 0.9 WTE
- [ ] 1.0 WTE

Page 5: Ultrasound Training and Mentorship

5. What ultrasound training have you undertaken? (please select all that apply) NB CASE = Consortium for the Accreditation of Sonographic Education ✱ *Required*

- ☐ None
- ☐ Pre-registration degree programme
- ☐ On-line
- ☐ In house (e.g. with a clinical mentor)
- ☐ Ultrasound machine manufacturers course
- ☐ Organisation led course (e.g. British Society for Rheumatology)
- ☐ CASE accredited focus or short course
- ☐ CASE accredited full course (e.g. Post-Graduate Certificate or Diploma)
- ☐ Other

5.a. If you selected Other, please specify:

5.b. How long ago did you complete your pre-registration degree programme? Please state (years)

5.c. How long ago did you complete your on-line training? Please state (years)

5.d. How long ago did you complete your in house (e.g. with a clinical mentor) training? Please state (years)

5.e. How long ago did you complete your ultrasound machine manufacturers course training? Please state (years)

5.f. How long ago did you complete your organisation led course (e.g. British Society for Rheumatology) training? Please state (years)

5.g. How long ago did you complete your CASE accredited focus or short course training? Please state (years)

5.h. How long ago did you complete your CASE accredited full course (e.g. Post-Graduate Certificate or Diploma) training? Please state (years)

5.i. How long ago did you complete your other training? Please state (years)

6. Do you have a current ultrasound mentor? ✱ *Required*

- Yes
- No

6.a. If yes, please state which profession your mentor(s) is(are) from.

6.b. Would you like an ultrasound mentor to support your practice?

- Yes
- No

6.b.i. If yes, in what area of ultrasound practice would you like mentorship? (please select all that apply)

- Ultrasound Imaging
- Vascular hand-held Doppler ultrasound
- Therapeutic ultrasound
- Other

6.b.i.a. If you selected Other, please specify:

7. Have you previously or are you currently providing ultrasound mentorship for other Podiatrists? ✱ *Required*

- Yes
- No

7.a. If yes, please select all the areas that this applies to.

- ☐ Ultrasound imaging
- ☐ Vascular hand-held Doppler ultrasound
- ☐ Therapeutic ultrasound
- ☐ Other

7.a.i. If you selected Other, please specify:

Page 6: End of the Survey

Thank you for taking the time to complete this survey.

The College of Podiatry Ultrasound in Podiatry Network aims to provide an opportunity to support podiatrists in all areas of clinical (e.g. musculoskeletal, diabetes and vascular) and surgical practice to undertake ultrasound training as well as those who are already using ultrasound in their practice.

If you would like more information or would like to join the Ultrasound in Podiatry Network please contact Dr Heidi Siddle h.siddle@leeds.ac.uk

---
